# Quasispecies Spatial Models for RNA Viruses with Different Replication Modes and Infection Strategies

**DOI:** 10.1371/journal.pone.0024884

**Published:** 2011-09-19

**Authors:** Josep Sardanyés, Santiago F. Elena

**Affiliations:** 1 Instituto de Biología Molecular y Celular de Plantas, Consejo Superior de Investigaciones Científicas, València, Spain; 2 The Santa Fe Institute, Santa Fe, New Mexico, United States of America; Albert Einstein College of Medicine, United States of America

## Abstract

Empirical observations and theoretical studies suggest that viruses may use different replication strategies to amplify their genomes, which impact the dynamics of mutation accumulation in viral populations and therefore, their fitness and virulence. Similarly, during natural infections, viruses replicate and infect cells that are rarely in suspension but spatially organized. Surprisingly, most quasispecies models of virus replication have ignored these two phenomena. In order to study these two viral characteristics, we have developed stochastic cellular automata models that simulate two different modes of replication (geometric vs stamping machine) for quasispecies replicating and spreading on a two-dimensional space. Furthermore, we explored these two replication models considering epistatic fitness landscapes (antagonistic vs synergistic) and different scenarios for cell-to-cell spread, one with free superinfection and another with superinfection inhibition. We found that the master sequences for populations replicating geometrically and with antagonistic fitness effects vanished at low critical mutation rates. By contrast, the highest critical mutation rate was observed for populations replicating geometrically but with a synergistic fitness landscape. Our simulations also showed that for stamping machine replication and antagonistic epistasis, a combination that appears to be common among plant viruses, populations further increased their robustness by inhibiting superinfection. We have also shown that the mode of replication strongly influenced the linkage between viral loci, which rapidly reached linkage equilibrium at increasing mutations for geometric replication. We also found that the strategy that minimized the time required to spread over the whole space was the stamping machine with antagonistic epistasis among mutations. Finally, our simulations revealed that the multiplicity of infection fluctuated but generically increased along time.

## Introduction

The dynamics and evolution of RNA virus populations is a current and important topic of research because RNA viruses are the most abundant pathogens of bacteria, humans and plants [1]. The role of these pathogens as a source of new emerging infectious diseases is also a very important subject of research in Virology and Epidemiology. RNA viruses present high population diversities, that with more or less precision are described in the virological literature as quasispecies [2]–[5]. A quasispecies can be roughly defined as a master sequence surrounded by a cloud of mutant genomes at the mutation-selection balance. Such a complex and polymorphic population structure may arise because of the large number of replication rounds that take place during intracellular amplification associated with the high mutation rates of the viral RNA-dependent RNA-polymerase owed to their lack of proof-reading activity [6]–[8]. Due to these peculiarities, RNA viruses have also served as excellent models for experimentally addressing important questions in evolutionary biology [9]–[11]. Several works on theoretical quasispecies [12]–[20] have been developed to understand key phenomena in virus dynamics and evolution. The convergence between theoretical and experimental results about virus dynamics and evolution is pivotal for the advance and success of future antiviral strategies [1], [21].

Although new insights for theoretical quasispecies can be extracted from nonlinear dynamical models, bifurcation theory or statistical physics [12], [20], [22], models usually take assumptions or simplifications that jeopardize experimental validation. In this sense, a very common assumption of viral quasispecies models has been that replication follows a geometric scheme. However, empirical data suggest that viral replication may strongly depart from this model (see below). The main goal of the present work is to study differential replication modes for RNA viruses incorporating other relevant features of viral infections, such as spatial structuring of host cells, epistasis among mutations and different mechanisms of infection. The consideration of all these features into a single model framework, and especially, the consideration of differential modes of replication, may cover the gap of previously existing models. A second common assumption of theoretical quasispecies involves oversimplified and unrealistic fitness landscapes. Similarly, the consideration of determinism, or the analysis of mean field models, which do not incorporate the effect of spatial correlations, has been of common practice. The latter assumption may pose serious constraints to the interpretation of results about real viral populations replicating in spatially structured host cells, like occur in plant or animal tissues. Empirical observations suggest strong spatial structuring of different genotypes in different areas of a leaf [23]–[25] or different parts of the plant [26], [27]. Similarly, the analysis of multiple samples from different tissues suggest that many animal viruses differentiate in tissue-specific subpopulations [28]–[30]. Broadly speaking, it is known that spatial correlations can influence the dynamics of nonlinear dynamical systems [20], [31], [32]. The effect of space on quasispecies dynamics has been investigated in several works. For example, limited diffusion was shown to provide mutant classes with a competitive advantage, also decreasing the critical mutation rate, 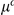 (i.e., the mutation rate beyond which the mutant genomes outcompete the master sequence) at which the error threshold phase transition occurs [14], [17]. The effect of space in the competition dynamics of two quasipecies has been also studied in the context of the survival of the flattest effect [20]. More recently, the effect of spatial competition on the diversity for structured quasispecies has been investigated by Aguirre *et al.* [19].

During the earlier stages of infection by plant viruses, the spreading of the viral population within a host starts from the initially infected cells to the nearest neighbors through the plasmodesmata, in a process known as cell-to-cell movement. Although some studies on different viruses infecting their hosts show that systemic movement can cause strong population bottlenecks and highly heterogeneous viral subpopulations in different organs [27], [33]–[36], the effects of the population bottlenecks during cell-to-cell movement have not been deeply studied. In this context, a key parameter in virus evolution is the number of virus genomes infecting a given cell, a parameter known as the multiplicity of infection (MOI). MOI is important as it determines processes such as the rate of genetic exchange among genomes, selection intensity on viral genes, epistatic interactions, and the evolution of multipartite viruses [37], [38]. Several models of virus evolution have explored the role of MOI in host-pathogen interactions [38]–[42], but experimental estimations of MOI along infection are still scarce, and only a handful of studies have estimated MOI in bacteriophages [43]–[45] and in the larvae of insects [46]. In the recent years, plant virologists have turned their attention to this problem. In a seminal study, González-Jara *et al.* [47] have obtained estimates of MOI for the *Tobacco mosaic virus* (TMV) infecting *Nicotiana benthamiana* plants. They followed the process of infection and characterized the temporal variation of MOI for two TMV genotypes, finding that MOI decreased as infection progressed. These authors suggested that such a reduction in MOI could be explained by mechanisms limiting superinfection and/or by genotype competition. More recently, Gutiérrez *et al.* have provided a spatio-temporal monitoring of the cellular MOI for the *Cauliflower mosaic virus* (CaMV) [48]. This second study revealed the presence of dynamic changes of MOI throughout the infectious cycle in the plant, with a maximum MOI reached at intermediated times post infection.

Theoretical and computational quasispecies models have mainly considered RNA populations replicating exponentially, more generally, geometrically (hereafter GR). In the case of single-stranded RNA viruses, GR implies that both the genomic and antigenomic viral strands are used as templates for replication, and thus the accumulation of mutations is large because mutant genomes also serve as templates for replication. From this replication mode, the distribution of the number of mutants per infected cell follows the Luria-Delbrück distribution [49]. Experimental studies carried out with bacteriophage 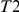 supported such strategy [50]. Alternatively, viruses may replicate according to the stamping machine replication mode (SMR). Under this scenario, the initially infecting genomic strand is used for the production of one or few antigenomic ones, which are then used as templates for the generation of all the progeny of positive-sense strands that will then be encapsidated to continue the infection process. In this case, the number of mutant genomes per infected cell follows a Poisson distribution. Such a distribution of mutants was found for phage 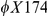 [52]. Intermediate modes of replication, where some fraction of positive-sense strands may be also replicated, have been described for phage 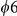, whose distribution of mutants slightly differed from the Poisson distribution [53]. Recently, the mode of replication was inferred for *Turnip mosaic virus* (TuMV) [51], which was largely dominated by the SMR. The role of the replication mode in the accumulation of deleterious mutations as well as in the mutational robustness of well-mixed quasispecies populations was recently investigated by Sardanyés *et al.* [54]. These authors developed theoretical and computational models to characterize the effect of the replication mode on the accumulation of mutations for positive-sense, single-stranded RNA viruses under different fitness landscapes, paying especial attention to the epistatic fitness landscape, which has been confirmed in several examples of RNA vuses (see [55] for a review). In short, the main conclusion of this study was that the SMR was less sensitive to the effect of mutations and compatible with higher critical mutation rates.

The aim of the present work is to extend the results of [54] by incorporating the effect of space. Theoretical or computational works exploring the effects of the mode of replication on RNA virus dynamics are scarce, and previous attempts to tackle this question have not considered spatially-distributed viral populations [54], [56]. The question we are addressing in this study is precisely what is the effect of space for viral quasispecies replicating under GR and SMR. To do so, we have developed stochastic cellular automata (CA) simulation models that consider replication, mutation and cell-to-cell infection in a two-dimensional environment. As we did in [54], here we also take a very general modeling approach simulating single-stranded RNA viral populations *in silico*, using digital quasispecies. Hence, the results of our study might serve as an approach to the dynamics of RNA viruses like arteriviruses, picornaviruses, flaviviruses or togaviruses, among others. Finally, our modeling approach also allows us to explore two different infection strategies that have been widely observed in experiments with viruses, namely, free superinfection (i.e., already infected cells are susceptible to additional infections) and superinfection exclusion (i.e., viruses of infected cells block the entrance of new viruses) [57]–[59]. Although our model is still a simplified picture of real viral infections, it represents a major step forward from previous models since it incorporates key features of real viral populations (e.g., different replication modes and different infection strategies).

## Simulation Model

**Figure 1:**
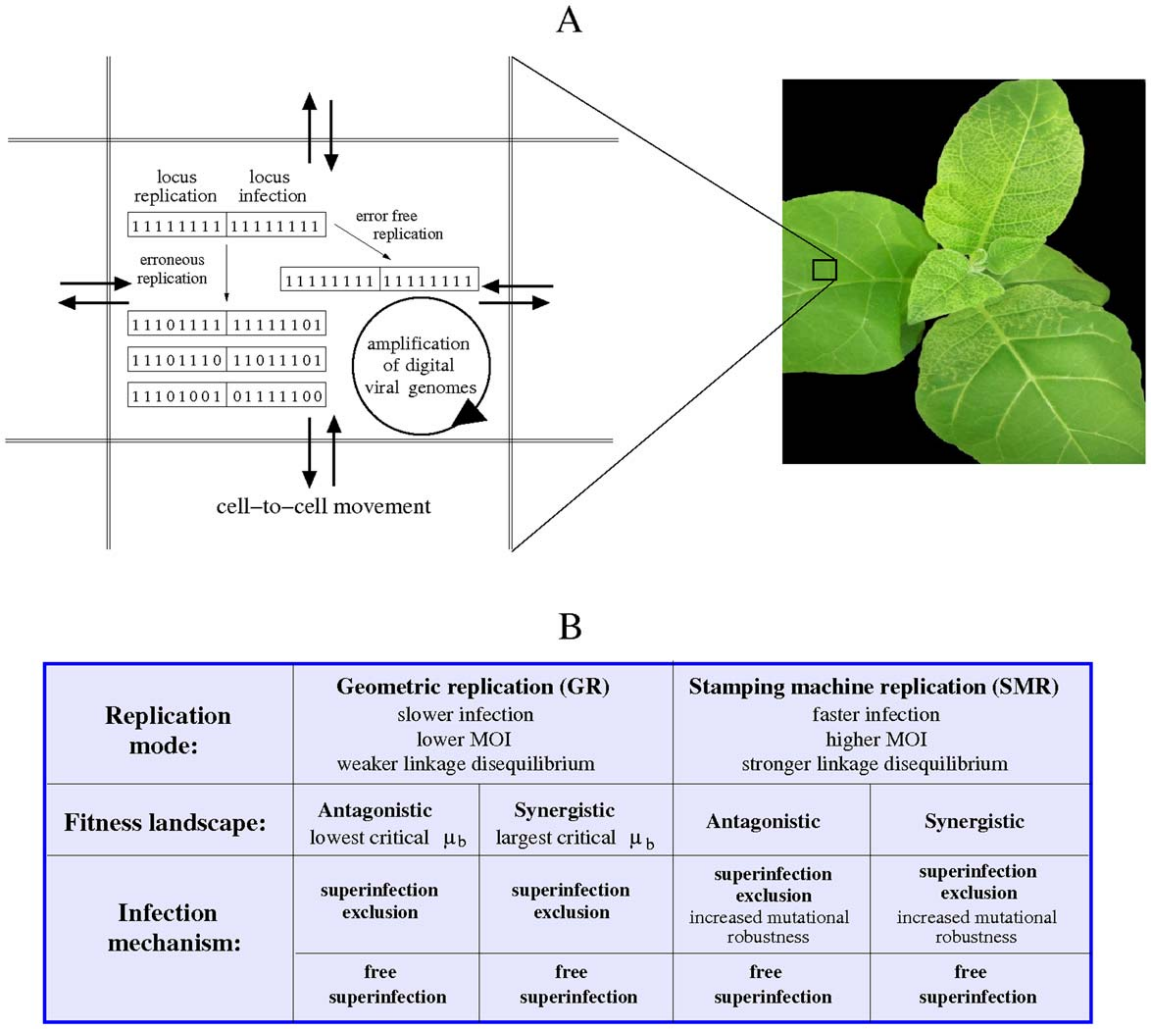
Schematic model description and scenarios analyzed. (A) Rules implemented in the CA model simulating *in silico* intracellular viral replication and cell-to-cell movement causing leaf infection. The bit strings replicate inside each of the lattice cells following geometric replication (GR) or stamping machine replication (SMR). When the quasispecies achieves its maximum population size inside a cell, a given string within that cell can move towards a neighboring one starting a new infection. The fitness of each string is considered as the probabilities of replication and infection, encoded in two different loci. The photograph corresponds to a plant of *Nicotiana tabacum* with some leaves infected by the positive-sense RNA virus *Tobacco etch virus*. (B) Table showing all the scenarios studied with the CA model summarizing the most relevant results. These include the mode of replication, epistatic fitness landscapes and two different mechanisms of viral infection.

The effect of the replication mode (geometric replication, GR; and stamping machine replication, SMR) in the spatial dynamics of replication and infection of a quasispecies is studied by using stochastic cellular automata (CA) models. The CA works on a square state space 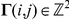, with 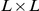 cells (we use 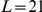) and zero-flux boundary conditions simulating the bounded system, for examle, of plant leaves (Figure 1A). Following the approach of Leuthäusser [60], [61], we use a bit-string description of the quasispecies population structure [20], [54], [62], [63]. Hence, we do a mapping between RNA sequence, defined as a chain of nucleotides involving a four-letter alphabet 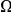, and a binary sequence, given by: 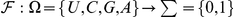. In this case, the strings contain a sequence of purines or pyrimidines that only incorporate the linear information encoded in the genotype. With such an approach we can analyze the spatial dynamics of RNA viruses using digital genomes and taking into account the mode of replication, mutation and cell-to-cell infection.

Each lattice cell in 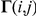 has the potential to contain a maximum population of 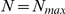 sequences (we use 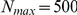). That is, each cell has 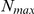 sites which can be occupied by newly produced strings or by strings entering from neighbor cells during cell-to-cell movement. Each one of these sequences, 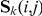, with 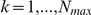, is a small digital genome of length 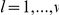 (we use strings of length 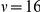 bits) i.e., 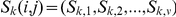, representing a vertex 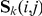, of a discrete, 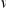-dimensional sequence space (hypercube, 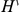): 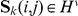, living in the 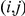 lattice cell (Figure 1A). Hence the total population of strings once the lattice is full is 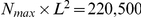 strings, being 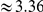-fold the number of different strings of the entire sequence space. In order to model the effect of mutations in the fitness associated to the replication process as well as to infection, we consider that the digital genomes contain two different loci, each of them with a length of 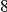 bits. These two loci will be used to assign the fitness of each genome tied to the processes of replication and cell-to-cell infection. By doing so, we decouple replication from transmission: a genome may be able of replicating but not of transmiting itself, and viceversa. As a first approach and for the sake of simplicity, we make a direct mapping between the genotype and both infection and replication success of a given string, without explicitly modeling the production of replicase or movement proteins. Moreover, our model also obviates recombination and/or complementation between different genotypes. For both replication modes we study two different deleterious fitness landscapes with epistatic interactions (Figure 1B). We specifically study the antagonistic fitness landscape, as being the more commonly described in RNA viruses [64]–[67], in which the deleterious effect of multiple mutations together is lower than expected from their individual effects. For the sake of completeness, we also implemented a synergistic landscape, in which an increasing number of mutations has a stronger deleterious effect than expected from the effects of each individual mutation. The fitness function associated to replication success, 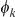, for the 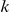-th sequence is given by:
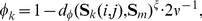
(1)where 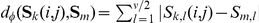. The fitness associated to infection success, 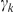, for the 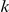-th sequence is given by:
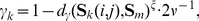
(2)here with 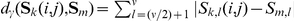. For both cases, 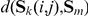 is the Hamming distance between each locus of the 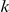-th sequence and the corresponding locus of the master sequence (i.e., the all-ones string labeled 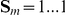). Note that 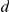 computes the number of mutations of the 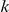-th string (i.e., number of bits 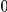). The parameter 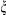 denotes the sign and the strength of epistasis (see [54], [55]). For the antagonistic landscape we use 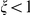, while for the synergistic one we will use 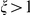. The model does not explicitly include beneficial mutations, but, according to the studied landscapes, backward mutations will always involve a fitness increase.

The CA works as follows: we first choose a random cell of the lattice whenever the lattice is not full. If the chosen cell is empty, we continue with the same process. However, if the chosen cell is not empty, then we consider that a generation has taken place. Then, for such a cell and generation time, we first apply 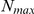 times the rule of intracellular replication in order to ensure that, on average, all the strings inside the chosen cell are updated once per generation. After the 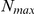 rounds of replication we apply the rule of cell-to-cell infection. These two rules are applied until the whole lattice is full of strings. Next, we describe the state-transition rules of the CA.

1. **Intracellular replication.** We choose at random a replicating string of the cell which is copied with a probability proportional to its fitness 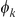 into another randomly chosen empty site. Although we do not explicitly consider the polarity of the strings, we can differentiate between GR and SMR. On the one hand, GR is implemented by considering that all the strings will replicate proportionally to their replicative fitness, 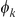, giving rise to more replicating genomes. On the other hand, SMR is implemented as follows: the strings belonging to the initial conditions or to the newly infecting strings entering into the cell (assumed to be positive-sense strands) will replicate only once, giving place to the template genomes that will be used for further replication. Such initial strings will not continue replication in the following generations. Instead, their offspring (acting as antigenomic templates) will continue replicating, giving rise to non-replicating genomes. By doing this, the progeny of strands will only be generated from the templates synthesized from the first infecting genomes. Replication mechanism presents per-bit mutation probability, 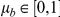, per replication cycle.
2. **Cell-to-cell infection.** We will assume that the strings within a cell can infect neighboring cells when 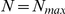. When this condition is fulfilled, we choose a string at random inside that cell, which moves with probability 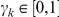 to a randomly chosen empty site of a neighboring cell (we use a von Newmann neighborhood i.e., 4 nearest neighbours). We will also consider that all the strings infecting neighboring cells will become replicators independently if they were previously replicators or not. However, in order to simulate a more realistic scenario for positive-sense RNA viruses (where the genomic strands are encapsidated to infect neighboring cells), we will assume that for GR a given string will infect the neighboring cell with probability 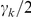, because, on average, GR is producing the same number of genomic and antigenomic strands (i.e., 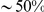 of each type of string). On the contrary, for SMR, we will take into account that all the strings can infect neighboring cells with probability 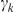 because the majority of the offspring are positive-sense strands. For both replication modes and fitness landscapes, we will study two possible mechanisms of infection (see Figure 1B): (i) *superinfection exclusion* (SE) and (ii) *free superinfection* (FS). In case (i) we consider that when a string of a given cell, e.g., 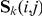, infects an empty neighbor or a neighbor that has not reached yet 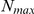, i. e., 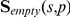 with 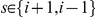, 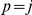; and 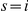, 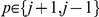, the cell 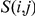 cannot be infected again by strings from neighboring cells. In (ii) we will consider that, independently of their current infection status, a given cell can be infected by a new string from a neighboring cell.

The algorithm starts with the central cell of the lattice inoculated with an initial population of 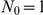 master sequences (assuming that are positive-sense strands). As previously mentioned, for GR these strings will always replicate producing the offspring that will also replicate. For SMR, they will replicate once, giving rise to templates that will be the responsible of producing the entire progeny of genomes that will not further contribute to the generation of more strands. We note that in our computational model there is only one real free parameter given by mutation rate.

## Results

### The lowest critical mutation rate, 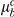, is found for GR and antagonistic epistasis

**Figure 2:**
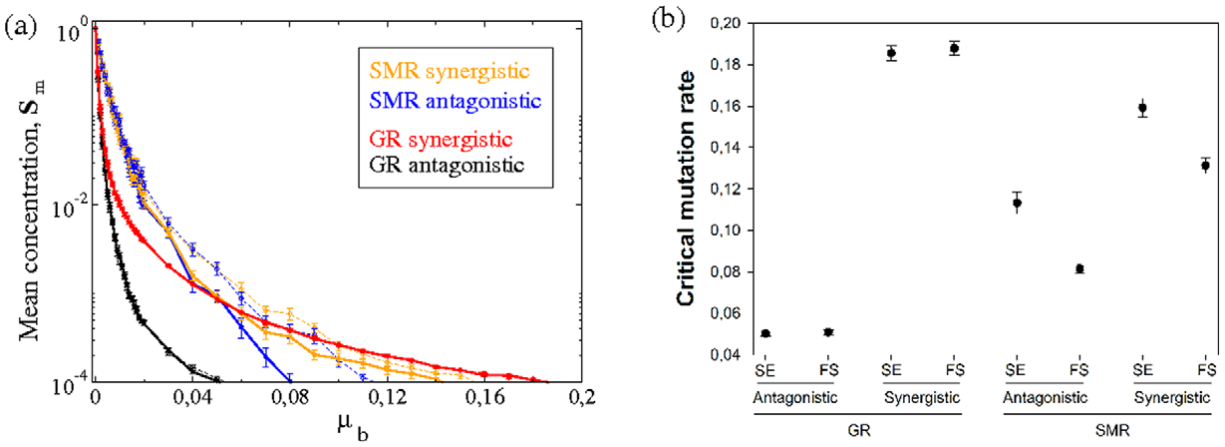
Differences in the error threshold and in the effects of mutation rate on the master sequence concentration. (a) Equilibrium concentrations for the master sequences, 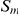, at increasing per-bit mutation rate, 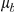. Here thin dashed lines correspond to superinfection exclusion (SE) and thick solid lines to simulations with free superinfection (FS). Each data point is the mean value (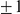 SEM) computed over 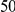 independent runs. (b) Mean critical mutation rates, 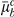 (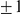 SEM), computed as the minimum mutation rate involving a mean concentration (computed over 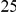 independent replicas) of master sequences lower than 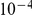. We study the values of 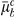 considering SE and FS, exploring both antagonistic (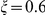) and synergistic (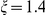) cases for the stamping machine replication (SMR) and geometric replication (GR) modes.

We first investigate the value of critical mutation rate per bit, 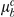, at which the population experiences the transition to the error threshold. To do so, we study how the concentration of master sequences changes at increasing mutation rates [Figure 2(a)]. The per-bit critical mutation rate, 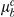 (i.e., the mutation rate beyond which the population of strings is dominated by mutants) is considered as the lowest value of mutation rate at which the concentration of master sequences is lower than 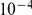. Although our model does not incorporate degradation of strings, the error threshold we are characterizing corresponds to extremely low population numbers of master sequences due to mutation processes. The numerical value attributed to the critical mutation rate involves an upper bound with a small population of master sequences (i. e., 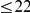 over 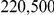). The increase in mutation rate involves a decrease of the master sequences for all the studied combinations. However, the magnitude of such a decrease strongly depends on the replication mode (Figure 2(b), ANOVA main effect 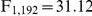, 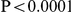) and on the type of the fitness landscape (Figure 2(b), ANOVA main effect 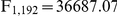, 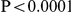). In Figure 2(a) we show the normalized mean concentration (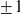 SEM) of master sequences in the whole lattice once is full computed over 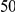 independent replicas. For the combination of GR and antagonistic fitness landscape the critical mutation rate shows the lowest value (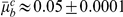). However, for the combination of GR with a synergistic fitness landscape the critical mutation rate drastically increases, taking values of 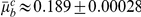. If we compare the effect of the fitness landscape for the SMR mode, similar results are found: the critical mutation rate is generally larger for the synergistic fitness landscape, independently of the infection strategy. However, the effect of the fitness landscape is not the same for both replication modes (Figure 2(b), ANOVA interaction term 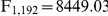, 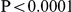). While the difference between synergistic and antagonistic landscapes on 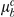 is, on average, 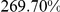 larger for GR, this difference drops to 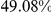 for SMR. Although previous results for well-mixed populations suggest that for SMR the population of master sequences might be less sensitive to mutation [54], the inclusion of spatial correlations allows the stable existence of master sequences at very high mutation rates provided the combination of GR and a synergistic fitness landscape. This phenomenon may be due to the effect of purifying selection, which under GR and the synergistic fitness landscape is more efficient because the production of strings with a very low or no fitness increases, and the competition for space is not so strong as for the SMR mode, where the master sequences are competing with higher fitness sequences and then are suffering the error threshold at lower values of mutation.

Next, we evaluate the effect that the infection strategy (FS vs SE) may have on 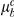. First, we find that, on average, SE is compatible with 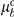 values that are 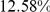 larger than if FS is allowed, being this difference highly significant (Figure 2(b), ANOVA main effect 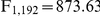, 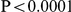). Second, this main effect depends on the replication mode (Figure 2(b), ANOVA interaction term 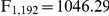, 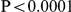). For GR, the increase in 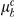 associated to SE is only 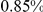 larger than for FS. By contrast, for SMR 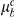 is 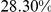 larger if superinfection is not allowed in the system. This differential effect can be rationalized as follows: if infection is limited and no strings can enter into a cell once the quasispecies has started infection to a neighboring cell, the population of master strings is more robust and can persist under larger mutation rates. On the other hand, if superinfection takes place, the critical mutation rate diminishes and the quasispecies enters into error catastrophe at lower values of 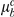. Third, in a lesser extent the effect of the infection strategy also depends on the topography of the fitness landscape (Figure 2(b), ANOVA interaction term 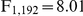, 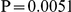). On average, by SE 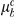 is 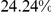 larger if the fitness landscape is antagonistic but only 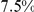 if the landscape is synergistic. No significant three-ways interaction has been detected (Figure 2(b), ANOVA three-ways interaction 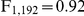, 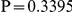).

### Dynamics and spatial distribution of digital quasispecies

**Figure 3:**
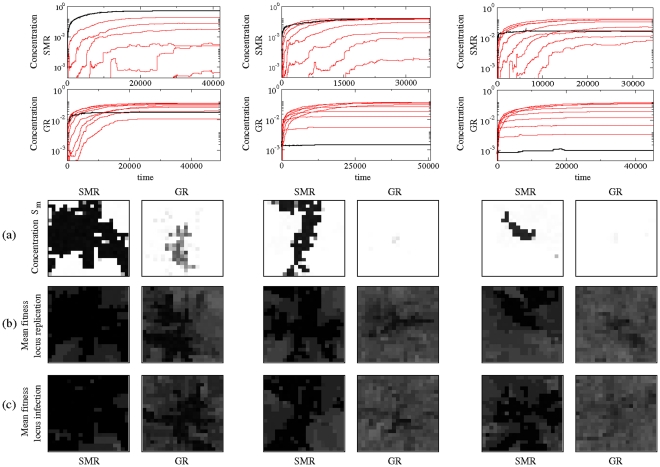
Time series and spatial patterns at increasing mutation rates. Spatio-temporal dynamics for the antagonistic fitness landscape (with 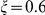) with superinfection exclusion, using (from left to right): 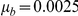, 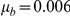 and 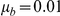. (Upper panels) Time series for the master sequence (thick black line) and the pool of mutants with 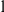 to 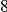 mutations (red lines). For each value of mutation we also show the spatial distribution of master genomes (a), and the mean fitness of replication (b) and infection (c) loci of the quasispecies once it has completely colonized the whole lattice [here, as well as in Figures 8, 9 and 10, the two-dimensional spatial patterns will be shown in a gray gradient. Values of zero concentration of the master sequence, 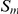, or zero-fitness are displayed in white, while maximum (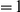) values of fitness or normalized concentrations are shown in black].

The space-time dynamics of the digital quasispecies has also been investigated for all combinations of fitness landscapes, replication modes and infection strategies. For illustrative purposes, Figure 3 shows the results obtained for the antagonistic fitness landscape considering SE. The other cases are shown as supplementary material (Figure S1 antagonistic landscape with FS, Figure S2 synergistic landscape with SE, and Figure S3 synergistic landscape with FS). As expected, for both replication modes, an increase in mutation rates involves a decrease of the concentration of the master genomes in all the studied cases once all lattice cells are totally full. Such decrease is much more accentuated for the GR mode. Generically, the master sequences can persist with SMR. However, for the GR mode, the master sequences maintain very low numbers even for small values of mutation rate (i.e., 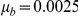). The most important differences between fitness landscapes correspond to the spatial distribution of the fitness of the quasispecies in the lattice. For both fitness landscapes we show that the mean fitness of both loci per cell is lower for the GR mode, while for the SMR mode the mean fitness per cell displays darker gray colours, being nearer to the maximum value (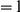, in black). Due to the nature of the fitness landscape we see that for the synergistic landscape, the mean fitness drastically reduces as depicted for the clearer spatial patterns shown in Figures S2 and S3, being much more pronounced for the GR mode. For the antagonistic landscape (with 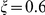) the value of minimum fitness per locus is 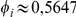.

### Increasing mutation for GR rapidly stabilizes digital quasispecies loci at linkage equilibrium

**Figure 4:**
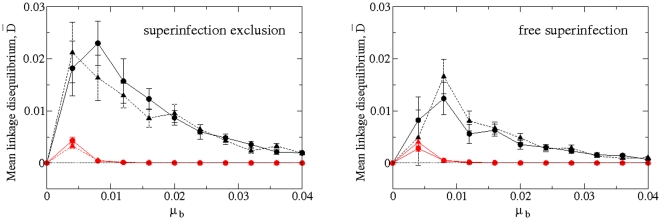
GR rapidly stabilizes digital quasispecies at linkage equilibrium at increasing mutation rates. Mean linkage disequilibrium, 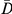 (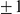 SEM) averaged over 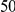 independent runs. We show two panels with SE (left) and FS (right). For each case: SMR (black) and GR (red), antagonistic epistasis (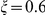, dashed line and triangles) and synergistic epistasis (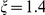, solid lines and circles).

Next we evaluate whether mutations at the two loci (i.e., replication and movement) associate randomly in the resulting viral population or linkage disequilibrium is created. Three forces may create linkage disequilibrium, namely mutation, selection and the sampling events that take place to initiate new cell infections. We are not intended to disentangling the contribution of these three mechanisms to the disequilibrium but just to determine wheter it may exist. To this end, we compute the linkage disequilibrium coefficient, 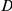, between two alleles of the two loci [68]. The alleles are differentiated for each locus, given by master (i.e., all-ones locus, indicated with 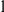) and mutant (i.e., a locus with one or more zeros, indicated with 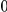) loci. Hence, 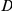 is computed from 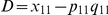, with 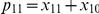 and 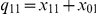. Here, 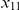 is the relative frequency of master sequences in the whole lattice, once the quasispecies has infected the whole space. The value of 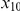 corresponds to the relative frequency of strings in the whole lattice with master replication locus and mutant infection locus and 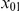 is the relative frequency of strings once the lattice is filled with mutant replication locus and master infection locus. We compute the mean linkage disequilibrium, 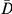, over 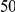 independent runs (after the lattice was completely full of strings), at increasing mutation rates for both replication modes also considering the antagonistic and synergistic landscapes for both infection strategies. The results, displayed in Figure 4 considering SE, clearly show that the main force determining the linkage disequilibrium is the mode of replication. For both replication modes, 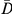 first increases reaching a maximum value and then declines at increasing mutation. However, in the entire range of mutation rates analyzed (i.e., 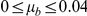), 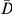 remains significantly larger than zero only for the SMR mode and regardless of whether superinfection was allowed or not. By contrast, for relatively low values of mutation rate, GR quickly reaches random association of alleles at both loci. This effect of the mode of replication can be explained by the fact that SMR produces genomes with a lower number of mutations than GR. By using already mutated templates, GR generates molecules carrying multiple mutations and thus, breaking any association between alleles at both loci.

Figure 4 also shows that the topography of the fitness landscape has a minor effect on 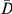, specially for the case of GR. For the case of SMR the effect depends on whether superinfection is allowed in the system. If SE is the norm, then the maximum linkage disequilibrium is reached at mutation rates larger for synergistic landscapes (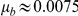) than for antagonistic ones (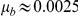). By contrast, if superinfection is allowed, the maximum is reached at the same mutation rate regardless the landscape topography (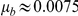). On average, the maximum value of 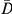 is 1.5-fold larger if SE exists than if superinfection occurs in a free manner.

### The multiplicity of infection generically fluctuates and increases along time as infection progresses

**Figure 5:**
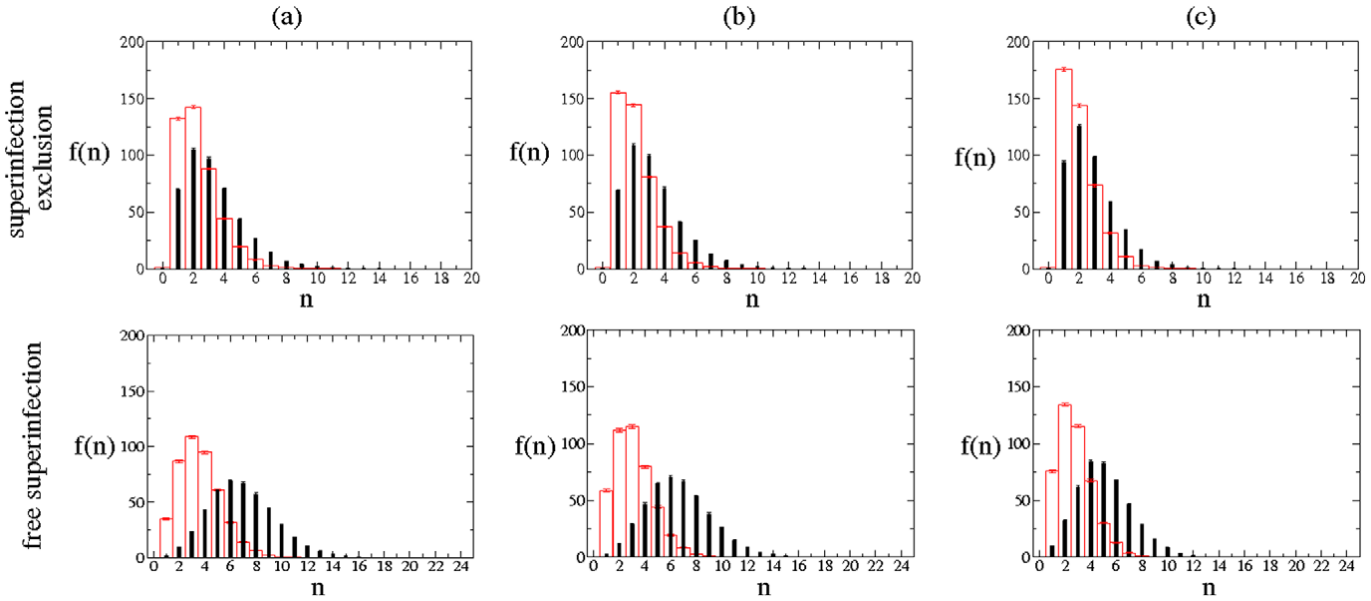
Dependence of the MOI on the mode of replication and on the infection strategy. Absolut frequency distributions, 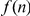, of the number of cells with 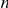 infections for the SMR (black histograms) and GR (red histograms) for the antagonistic fintess landscape (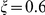) with (a) 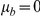, (b) 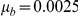 and (c) 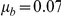. The histograms correspond to the average (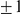 SEM) number of cells with 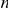 entering strings computed over 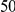 independent runs once the lattice is completely full of strings. In the upper and the lower row, we show the results with SE and FS, respectively.

Another interesting phenomenon that can be explored with our computational models is how MOI changes among replication modes for both fitness landscapes, as well as the spatial patterns of infections inside each cell of the lattice. As we mentioned in the Introduction, MOI is the number of viral particles infecting a host cell. Figure 5 shows the distribution of MOIs per cell for simulations with and without SE and for the case of antagonistic fitness landscapes (the results for the synergistic fitness landscape are shown in Figures S5 and S6 and are not discussed because are qualitatively similar to those shown in Figure 5). The frequency distribution of MOIs is computed once the whole lattice is completely full of strings. That is, after whole infection, we compute how many lattice cells are infected by 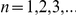, strings. In both cases the spatial distribution of infections is disordered, and no clear spatial patterns are found (Figure S4). Overall, for both fitness landscapes, the average number MOI per cell is higher for the SMR than for the GR.

To analyze in a more quantitative way the data shown in Figure 5, we fit MOI values to a generalized linear model. MOI is assumed to be Poisson distributed and the mode of replication and whether superinfection is allowed or not are treated as main factors whereas the mutation rate (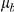) is incorporated in the model as a covariable. All interactions between factors and the covariable are included in the model. Overall, the mode of infection has a highly significant effect on MOI (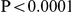), being it 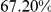 larger for SMR than for GR. As expected, allowing for FS makes MOI to increase up to 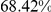 when compared to the case of SE (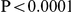). However, the effect of the mode of replication is not independent on the superinfection status. These two main factors interact in a very significant way (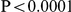), being the effect of the replication mode on MOI larger when superinfection is free (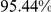) than when it is excluded (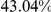). The covariable, 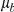, has a significant negative effect on MOI (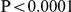). Increasing it in the range 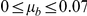 implies a decline in MOI of 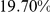. However, the magnitude of this decline depends on whether superinfection was free or limited (test of interaction term, 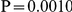), being smaller in the former situation (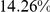) than in the latter (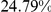). The effects of mutation rate and replication mode are independent (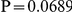). Similarly, the interaction between the two factors is not affected by 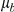 (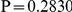).

**Figure 6:**
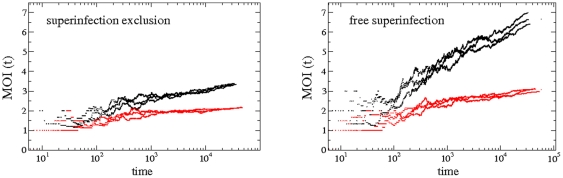
Time dynamics of the MOI during genomes infection. Time evolution of the multiplicity of infection, 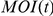 (in log-linear scale), computed from the number of genome entries over the total number of infected cells per generation time, represented with three trajectories for each replication mode (black: SMR and red: GR) for the antagonistic fitness landscape using 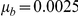. The results for SE are shown on the left and the simulations with FS are dislayed on the right.

Next, we explore the temporal dynamics of MOI. To do so, we compute, at each time generation, the mean number of strings that have entered into the cells of the lattice (averaged over all infected cells). The results are shown in Figure 6 for the antagonistic fitness landscape and 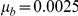 (no qualitative difference exists for the synergistic landscape). We specifically show two plots considering SE (left panel) and FS (right panel). For each case we display the time evolution of the 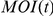 with three trajectories corresponding to three independent replicas. The quantity 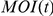 is a measure of how the lattice is filled along time, so it is a kind of cumulative measure which fluctuates because the data is normalized at each time generation by the total number of infected cells. The results of Figure 6 show that, independently of the replication mode and of the infection strategy, MOI fluctuates, but significantly increases as time progresses. Indeed, linear regression analyses confirm that the slope is significant in all four cases (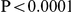 in all cases). An ANCOVA using mode of replication and superinfection status as factors and time as covariable shows that both factors have a significant effect on the average MOI reached in the lattice (in both cases 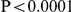). Interestingly, both factors show a significant interaction with the covariable (in both cases 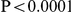), suggesting that the rate at which MOI increases with time depends on them. For instance, when superinfection is excluded, the slope of the regression line obtained for the SMR is 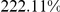 larger than when the virus replicative strategy is GR. This difference in the rates of MOI change among replicative strategies is even larger for the case of FS (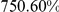), a difference supported by a significant three-ways interaction term in the ANCOVA (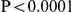). Therefore, we conclude that MOI increases with time but that the increase is faster if SMR is the replication strategy followed by the virus and if no SE mechanisms are at play. Qualitatively similar results have been found at increasing mutation rates and for the two fitness landscapes analyzed (data not shown).

The mean values of genome entries per cell once the lattice is completely full are represented, for each studied case, in Table 1 and 2. These results indicate that larger MOIs are found for the SMR mode under the antagonistic fitness landscape with superinfection. Generically, MOI decreases for the synergistic fitness landscape, for both SMR and GR modes. This phenomenon occurs when considering both SE and FS. We note that for both infection strategies and fitness landscapes, the values of MOI are always higher for the SMR mode. This result may reflect the implicit consideration of the sense of the strings (recall the assumption that for the GR the infection probability was halved because approximately the 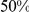 of the progeny might correspond to positive-sense strands). However, this is a consequence of the nature of the infections, since a single-stranded RNA virus might be able to encapsidate more genomic strands when replicating under SMR.

**Table 1:** Mean values (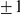 SEM) of the multiplicity of infection for the model with SE, computed over 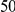 independent replicas once the lattice is completely infected by the quasispecies at increasing mutation rates, 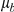, for both replication modes and both fitness landscapes (antagonistic with 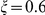; synergistic with 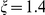).

	Stamping machine replication (SMR)		Geometric replication (GR)	
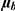	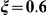	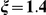	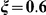	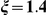
0	3.312 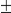 0.0105	3.322 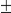 0.0102	2.355 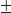 0.0792	2.330 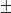 0.0080
0.0045	3.183 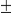 0.0119	3.176 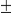 0.0147	2.119 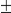 0.0077	2.001 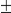 0.0070
0.0090	3.141 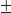 0.0118	3.055 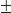 0.0166	2.077 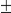 0.0064	1.935 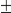 0.0089
0.0135	3.066 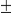 0.0124	2.950 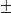 0.0148	2.032 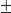 0.0059	1.894 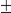 0.0073
0.0180	3.031 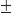 0.0134	2.891 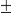 0.0181	2.044 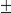 0.0079	1.899 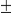 0.0069
0.0225	3.013 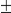 0.0115	2.854 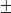 0.0137	2.027 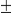 0.0069	1.883 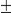 0.0069
0.0270	2.968 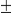 0.0091	2.807 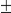 0.0159	2.036 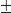 0.0071	1.882 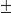 0.0075
0.0315	2.948 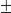 0.0118	2.782 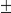 0.0162	2.031 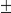 0.0078	1.872 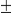 0.0056
0.0360	2.935 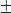 0.0120	2.740 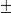 0.0170	2.027 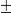 0.0071	1.891 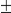 0.0070
0.0405	2.912 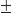 0.0099	2.720 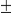 0.0149	2.038 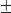 0.0072	1.883 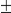 0.0082
0.0450	2.901 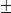 0.0112	2.685 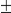 0.0171	2.034 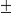 0.0073	1.889 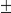 0.0071
0.0495	2.886 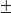 0.0096	2.687 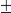 0.0152	2.040 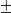 0.0082	1.910 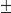 0.0066
0.0540	2.887 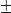 0.0118	2.666 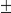 0.0540	2.021 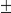 0.0057	1.892 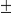 0.0043
0.0585	2.876 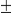 0.0088	2.670 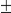 0.0150	2.025 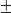 0.0086	1.895 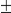 0.0059
0.0630	2.886 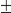 0.0098	2.654 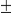 0.0124	2.042 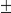 0.0058	1.908 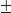 0.0059
0.0675	2.859 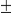 0.0087	2.625 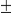 0.0139	2.025 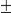 0.0069	1.905 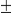 0.0076
0.0700	2.859 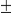 0.0099	2.624 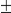 0.0134	2.029 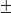 0.0070	1.912 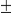 0.0081

**Table 2:** Mean values (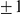 SEM) of the multiplicity of infection for the simulations considering FS, computed over 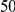 independent replicas once the lattice is completely infected by the quasispecies at increasing mutation rates, 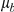, for both replication modes and both fitness landscapes (antagonistic with 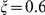; synergistic with 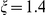).

	Stamping machine replication (SMR)		Geometric replication (GR)	
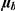	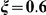	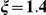	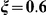	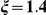
0	6.928 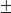 0.0291	6.969 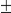 0.0269	3.556 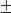 0.0129	3.582 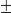 0.0116
0.0045	6.410 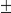 0.0373	6.336 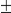 0.0393	2.967 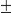 0.0133	2.736 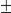 0.0199
0.0090	6.131 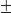 0.0329	5.779 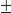 0.0577	2.826 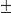 0.0123	2.477 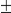 0.0124
0.0135	5.889 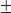 0.0280	5.549 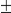 0.0396	2.818 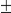 0.0106	2.374 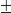 0.0127
0.0180	5.689 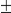 0.0355	5.255 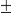 0.0479	2.785 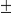 0.0097	2.293 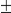 0.0112
0.0225	5.587 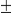 0.0328	4.900 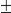 0.0432	2.767 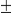 0.0135	2.273 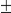 0.0097
0.0270	5.431 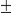 0.0264	4.748 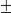 0.0475	2.781 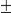 0.0112	2.213 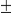 0.0188
0.0315	5.331 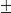 0.0313	4.581 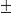 0.0428	2.798 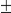 0.0117	2.198 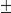 0.0109
0.0360	5.356 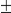 0.0237	4.578 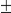 0.0370	2.777 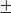 0.0115	2.173 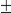 0.0083
0.0405	5.269 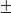 0.0244	4.407 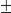 0.0340	2.751 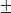 0.0127	2.163 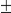 0.0106
0.0450	5.195 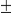 0.0239	4.324 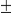 0.0343	2.755 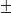 0.0113	2.160 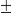 0.0094
0.0495	5.210 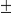 0.0210	4.284 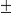 0.0342	2.769 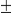 0.0109	2.151 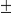 0.0082
0.0540	5.140 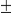 0.0198	4.188 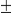 0.0301	2.766 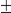 0.0114	2.153 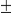 0.0094
0.0585	5.104 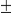 0.0207	4.150 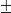 0.0352	2.769 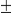 0.0127	2.145 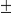 0.0084
0.0630	5.158 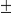 0.0206	4.061 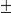 0.0321	2.751 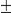 0.0100	2.159 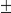 0.0109
0.0675	5.073 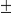 0.0247	4.003 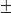 0.0254	2.778 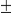 0.0117	2.150 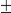 0.0104
0.0700	5.105 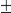 0.0229	3.985 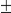 0.0284	2.748 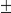 0.0114	2.150 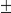 0.0088

### SMR together with antagonistic epistasis involves the fastest genomes colonization time

**Figure 7:**
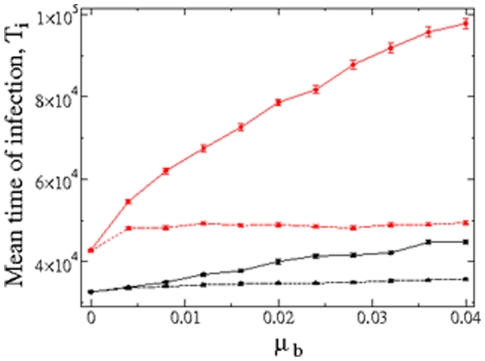
Efficiency of viral quasispecies in spreading and colonizing the whole lattice. Average time of infection, 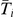, considering SE computed as the number of generations needed to fill the whole lattice for SMR (black) and GR (red). The results for antagonistic epistasis (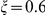) are shown with dashed line and triangles, while for synergistic epistasis (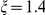) we use solid lines and circles. Each data point is the average (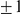 SEM) computed over 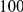 independent replicas.

Finally, as a measure of the performance of the different strategies in spreading the infection, we study the mean infection time, 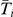, computed as the time it takes to complete the infection of all cells in the lattice (i.e., all cells contain 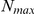 strings), for the two replicative strategies and fitness landscapes for increasing mutation rates but considering only the case of superinfection inhibition (Figure 7). As we did before, the data are fitted to an ANCOVA model in which landscape topography and replication mode are treated as fixed factors and 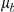 as covariable. A first result is that 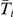 significantly increases with 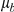 (test of covariable, 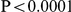). The mean infection times for the quasispecies replicating via the SMR are, for the whole range of mutation rates analyzed, systematically lower (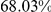, on average) than for quasispecies replicating via GR mode (test of replication mode main effect, 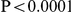). Hence, the spread of the strands is faster for the SMR mode, and such result is consistent independently of the fitness landscape assumed (test of the landscape topography main effect, 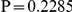; test of the interaction between landscape topography and replication mode, 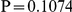). However, if one compares the time of infection between the two fitness landscapes for a given mode of replication we find that when mutations interact in a synergistic manner in determining fitness, the time required to complete an infection increases. This phenomenon is observed for both replication modes, although it is much more accentuated for the GR (test of the three-ways interaction term, 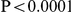). For this case, and for large mutation rates (e.g., 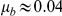), the mean time of infection for the synergistic landscape is 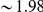-fold the time of infection for the antagonistic one. This may occur because for the synergistic landscape, mutations have a stronger deleterious effect and the quasispecies is producing less efficient mutants, who are not able to replicate and infect optimally, then needing a longer time to complete the infection of the whole lattice. For the combination of SMR and synergistic fitness landscape, the quasispecies also undergoes longer infection times as mutation rates grow, but such times remain always below the time needed for both landscapes under the GR mode. Under the SMR, the mean time of infection with 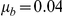 for the synergistic landscape is 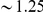-fold the one for the antagonistic landscape also under SMR. Finally, we also want to notice that for small mutation rates the values of 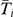 are quite similar for the SMR mode, but for the GR mode they rapidly diverge as 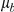 becomes larger (see. Figure 7 for details). Qualitatively similar results are found for the simulations run with FS (results not shown).

## Discussion

Different replication modes have been suggested for different viruses. Depending on whether genomes are replicated according to a stamping machine (SMR) or geometrically (GR), one may observe different compositions in the mutant spectrum of the quasispecies. For the SMR model, the initial antigenomic templates are the ones used for further replication, as observed for the phage 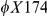 [52], where the number of mutants per infected cell followed a Poisson distribution. For the GR mode, such a distribution of mutants deviates from a Poisson, and follows a more complex distribution, termed Luria-Delbrück distribution. Experimental analysis showed that the bacteriophage 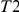 did not fit well a Poisson distribution, suggesting a GR strategy [50]. Intermediate modes of replication may also exist, as illustrated by experiments with phage 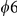 showing a distribution of mutants slightly different from the Poisson expectation [53], and with TuMV showing assymmetric accumulation of strands of both polarities [51]. Although the replication strategy for RNA viruses might have a deep impact in the accumulation of mutations and therefore in the fitness of the sequences, few works have explored the effects of different replication strategies in the population dynamics of viral quasispecies [54], [56]. Not to say that no work at all has been published exploring the interplay between the mode of replication and spatial correlations nor the existence of mechanisms of controlling superinfection. In this study we intend to cover the gap existing in biologically unrealistic models of virus replication and spread proposing new models that incorporate some of the most basic features of viral genomes. We analyzed the dynamics of replication and infection of quasispecies on a two-dimensional space by means of stochastic cellular automata models, especially focusing on the effect of different replication modes, topography of the fitness landscapes and existence of mechanisms inhibiting superinfection. Among the theoretical and computational approaches to study spatially-extended biological systems (e.g., metapopulation models, partial differential equations, etc.) we have chosen to simulate single-stranded RNA quasispecies by means of digital genomes using a cellular automaton approach. Such a strategy allows us to use spatial individual-based modeling taking into account the heterogeneous population structure of the quasispecies together with stochasticity. The digital genomes were constituted by two independent loci, one determining replication and the other determining the efficiency of cell-to-cell movement. Hence, our results may be useful to understand the dynamics and evolution of widely different viruses as far as they fulfill these basic assumptions. To the extend of our knowledge, our study is the first one simulating the spatio-temporal dynamics of single-stranded RNA viruses under different modes of replication. Moreover we modeled the fitness effects of mutations on each of these loci assuming two different epistatic fitness landscapes, one antagonistic and one synergistic. Supporting these choices, epistasis has been widely found in real RNA viruses [55], [69]. Together with the incorporation of two different replication modes and fitness landscapes in our simulations, we also investigated two possible infection strategies given by limitation of infection and superinfection, strategies known to occur in real viral population.

Non-spatial computational models with digital quasispecies showed that when replication proceeds via SMR, the population of master sequences is less sensitive to mutation and the critical mutation rates involved in the error threshold were always lower under GR, independently of the fitness landscape assumed [20]. In agreement with the previous work, our results show that the extinction of the master sequences occurs at a larger mutation rate with GR for the synergistic fitness landscape. However, in [20], the critical mutation rate of the quasispecies replicating under GR was always lower than the critical values under the SMR. In the simulations developed in this work we show that the largest critical mutation rate corresponds to the GR mode under synergistic interactions between mutations. This may be due to an enhanced synergy between space and purifying selection, where mutants with extremely deleterious mutations in both replication and infection loci could not spread over the lattice, favoring the selection of master sequences because reduced competition with other mutant sequences during replication and infection.

The analyses of two different infection strategies, given by superinfection exclusion (SE) and free superinfection (FS), revealed that when SE is considered, the critical mutation rates for SMR become larger, and thus the robustness of the quasispecies increased because the master sequence was able to persist for larger mutation rates. This result was in agreement with several works suggesting that when a cell is coinfected by different viral genomes, the fitness of individual genotypes may decrease in comparison with their fitness in a single infection due to competition processes [41], [70]. This phenomenon, however, seemed not to be important for the GR mode, probably because the accumulation of mutations was so large that the quasispecies in a given cell was dominated by the pool of mutants, and thus the entry of new mutants was irrelevant. Nevertheless, the entrance of new mutants in a cell for the SMR could drastically change the mutant spectrum of the progeny, especially if the infecting mutants carry many mutations.

Several examples of viruses that have evolved mechanisms to avoid superinfection have been reported and studied. For example, the wild-type 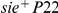 prophage is able to prevent growth of superinfecting phage 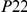 as well as of other phages like the 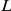, the 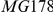 or the 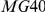, by means of exclusion mechanisms [71]. Other examples of viruses with mechanisms to avoid coinfection are the phage 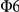 [43] as well as the *Vesicular stomatitis virus* (VSV) [57]. Whitaker-Dowling et al. [57], showed that the presence of mechanisms to limit superinfection is virus-dependent. They showed that infection of 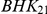 cells with either influenza viruses, *Encephalomyocarditis virus* or *Newcastle disease virus* did not inhibit superinfection by VSV, whereas cells initially infected with VSV were not susceptible for VSV superinfection. Our work suggests that a possible answer to the complex question of why some viruses limit superinfection but some others do not present such a property could rely on the mode of replication of each virus type. As previously mentioned, an important result we obtained in our simulations was that when replication proceeds via SMR, the limitation of superinfection largely affects the survival and maintenance of the master sequence, as a difference from the GR, where no significant differences were found between the two studied infection strategies.

The multiplicity of infection (MOI) (i.e., number of viral genomes infecting a host cell) is a key parameter in virus evolution because it can determine selection intensity on viral genomes, exchange among genomes or epistatic interactions. MOI has been mainly studied in different DNA and RNA bacteriophages [43]–[45]. However, very few experimental studies reporting estimates of MOI are found for virus infecting eukaryotic hosts. For example, MOI was studied for *Autographa californica nuclear polyhedrosis virus* during the infection of larvae of the lepidoptera *Trichoplusia ni* [46]. Only very recently, González-Jara *et al.* [47] and Gutiérrez *et al.* [48] have taken the task of studying the evolution of MOI during infection of plants by RNA viruses. In the former work, the authors carried out experiments of host colonization by two genotypes of the TMV infecting *N. benthamiana* plants. They found that MOI decreased during infection, and suggested that two nonexclusive processes could cause such a decrease: by mechanisms limiting superinfection and/or by genotype competition. Interestingly, our analyses of MOI dynamics showed that MOI fluctuated and that, independently of the replication mode, the fitness landscape and despite the existence of SE mechanisms, MOI increased in time. This finding suggests that the results found by González-Jara *et al.* [47] could not be explained by genotype competition or by SE. Hence, other mechanisms lowering MOI might be operating during infection. In the work of Gutiérrez *et al.* [48], the spatio-temporal dynamics of two competing variants of CaMV was monitored during the infection of turnip plants. They reported great changes of MOI at different infection phases during plant development [48]. Actually, our results, which might be interpreted as a quasispecies infecting a single tissue, reflected these fluctuations in MOI during the process of infection. A direct consequence of high MOI is the recombination and complementation between genotypes [72]–[74]. For the sake of simplicity, our models do not take into consideration both recombination and complementation processes between genotypes. These important phenomena, together with differential replication modes, should be considered in future research. Moreover, the consideration of a full model considering intracellular amplification and both cell-to-cell and systemic movement under the previous scenarios also remains a theoretical and a computational challenge.

Finally, a take-home message of our work is that important differences between non-spatial and spatially-structured models exist for quasispecies replicating under different replication modes. For instance, previous results indicated that the critical mutation rate of quasispecies was lower if replication was GR than if it was SMR independently of the unferlying fitness landscape [54]. However, our simulations have shown that by considering spatial correlations, the outcome would be the opposite for the synergistic landscape: GR would result in a more robust replication strategy. Moreover, we have also shown that mechanisms of superinfection exclusion during cell-to-cell movement might play an important role in virus robustness to mutations. Our findings also suggest that other mechanisms beyond limiting superinfection and/or genotype competition should be considered to explain the decrease in MOI reported in [47].

## Supporting Information

Figure S1
**Spatio-temporal dynamics for each mode of replication (stamping machine replication, SMR; geometric replication, GR) for the antagonistic fitness landscape (with** 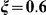 **) with free superinfection (FS), using (from left to right):** 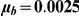 **,** 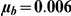 **and** 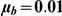 **.** (Upper panels) Time series for the master sequence (thick black line) and the pool of mutants with 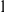 to 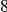 mutations (red lines). For each value of mutation we also show the spatial distribution of master genomes (a), and the mean fitness of replication (b) and infection (c) loci of the quasispecies [the spatial patterns will be shown in a gray gradient. Values of zero concentration of the master sequence, 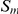, or zero-fitness are displayed in white, while maximum (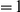) values of fitness or normalized concentrations are shown in black].(TIFF)

Figure S2
**Same as in the previous figure now for the synergistic fitness landscape with** 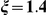 **and superinfection exclusion (SE) using the same mutation rates analyzed in the previous figure.**
(TIFF)

Figure S3 **Same as in the previous figure also for the synergistic fitness landscape but now considering FS.**
(TIFF)

Figure S4
**Spatial distribution of the number of infections,** 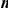 **(z-axis), in the lattice** 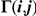 **for a single run under the antagonistic fitness landscape, using (from left to right): (a)** 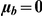 **, (b)** 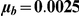 **and (c)** 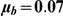 **.** We show the spatial pattern for SMR and GR. In the upper and in the lower two rows, we show the spatial patterns considering SE and FS, respectively. Note that these analyses show how does the multiplicity of infection (MOI) dependes on the mode of replication mode and on the fitness landscape, as well as how it distributes in the space.(TIFF)

Figure S5
**(Upper first row) Absolut frequency distribution,** 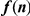 **, of the number of cells with** 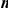 **infections for the SMR (black histograms) and GR (red histograms) for the synergistic fintess landscape (** 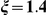 **) with SE.** Here (a) 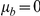, (b) 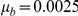 and (c) 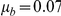. The histograms correspond to the average (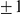 SEM) number of cells with 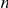 entering strings computed over 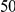 independent runs. (Lower two rows) Spatial distribution of the number of infections, 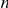 (z-axis), in the lattice 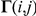 for a single run for each mutation rate used in (a). We show the results for SMR (upper spaces) and GR (lower spaces).(TIFF)

Figure S6
**Same as in the previous figure for the synergistic landscape with** 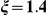 **and FS.**
(TIFF)
