# Supplementary figures and images for: Quasispecies Spatial Models for RNA Viruses with Different Replication Modes and Infection Strategies
Source: PLoS One. 2011 Sep 19;6(9):e24884. doi: 10.1371/journal.pone.0024884 (PMC3176287; doi:10.1371/journal.pone.0024884)

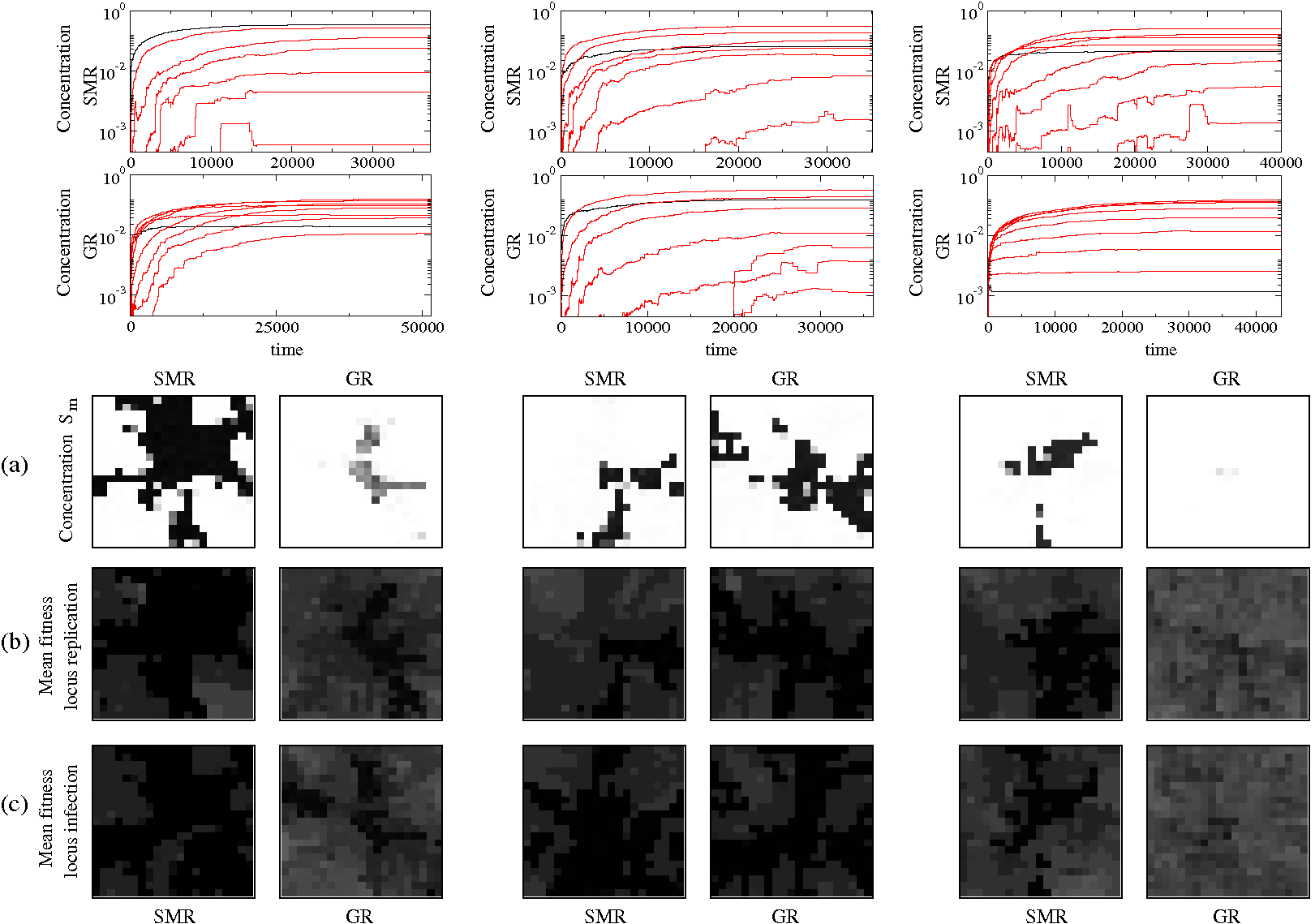

Supplement: Figure S1 — Spatio-temporal dynamics for each mode of replication (stamping machine replication, SMR; geometric replication, GR) for the antagonistic fitness landscape (with ) with free superinfection (FS), using (from left to right): , and . (Upper panels) Time series for the master sequence (thick black line) and the pool of mutants with to mutations (red lines). For each value of mutation we also show the spatial distribution of master genomes (a), and the mean fitness of replication (b) and infection (c) loci of the quasispecies [the spatial patterns will be shown in a gray gradient. Values of zero concentration of the master sequence, , or zero-fitness are displayed in white, while maximum () values of fitness or normalized concentrations are shown in black]. (TIFF) [file pone.0024884.s001.tiff]

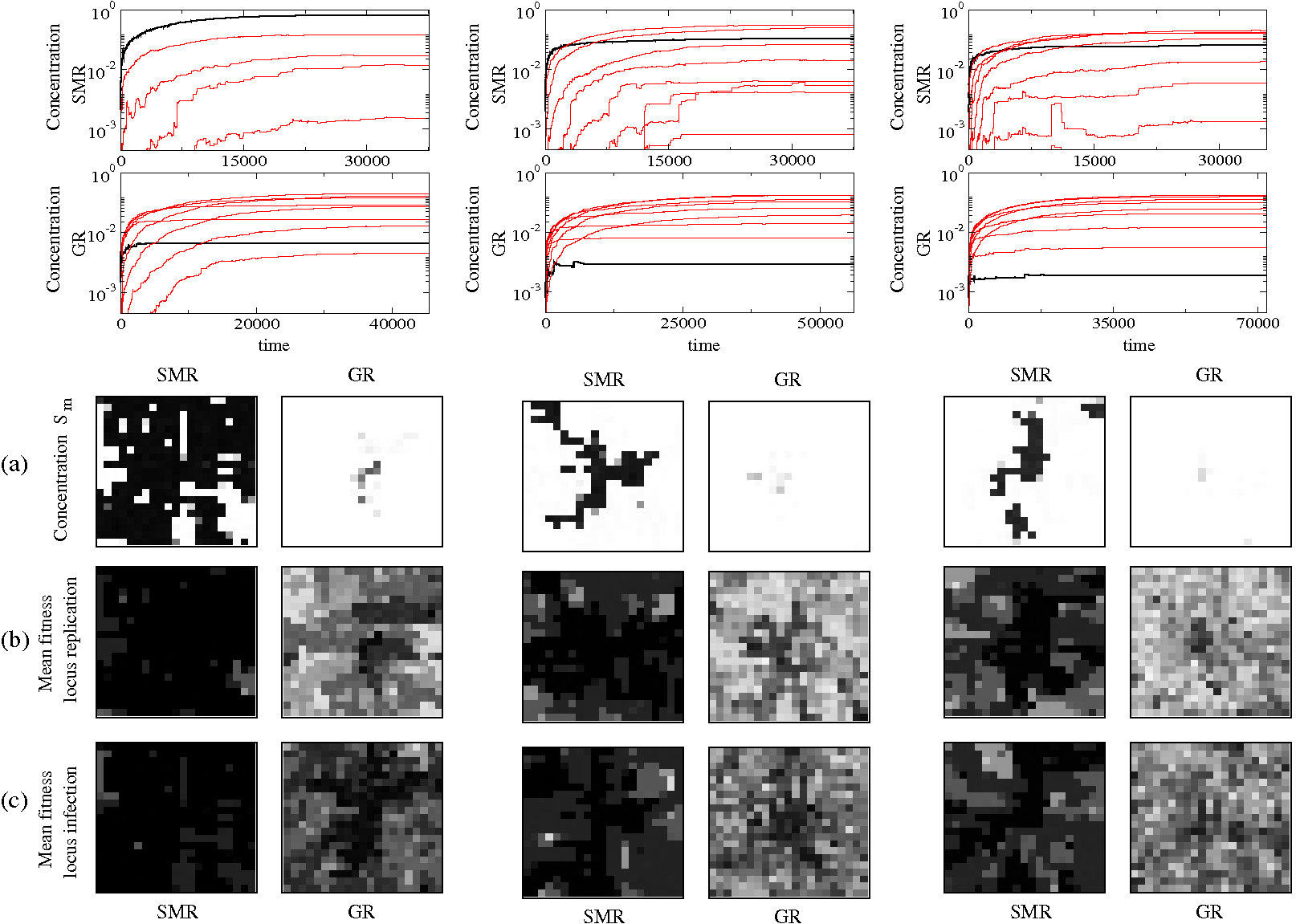

Supplement: Figure S2 — Same as in the previous figure now for the synergistic fitness landscape with and superinfection exclusion (SE) using the same mutation rates analyzed in the previous figure. (TIFF) [file pone.0024884.s002.tiff]

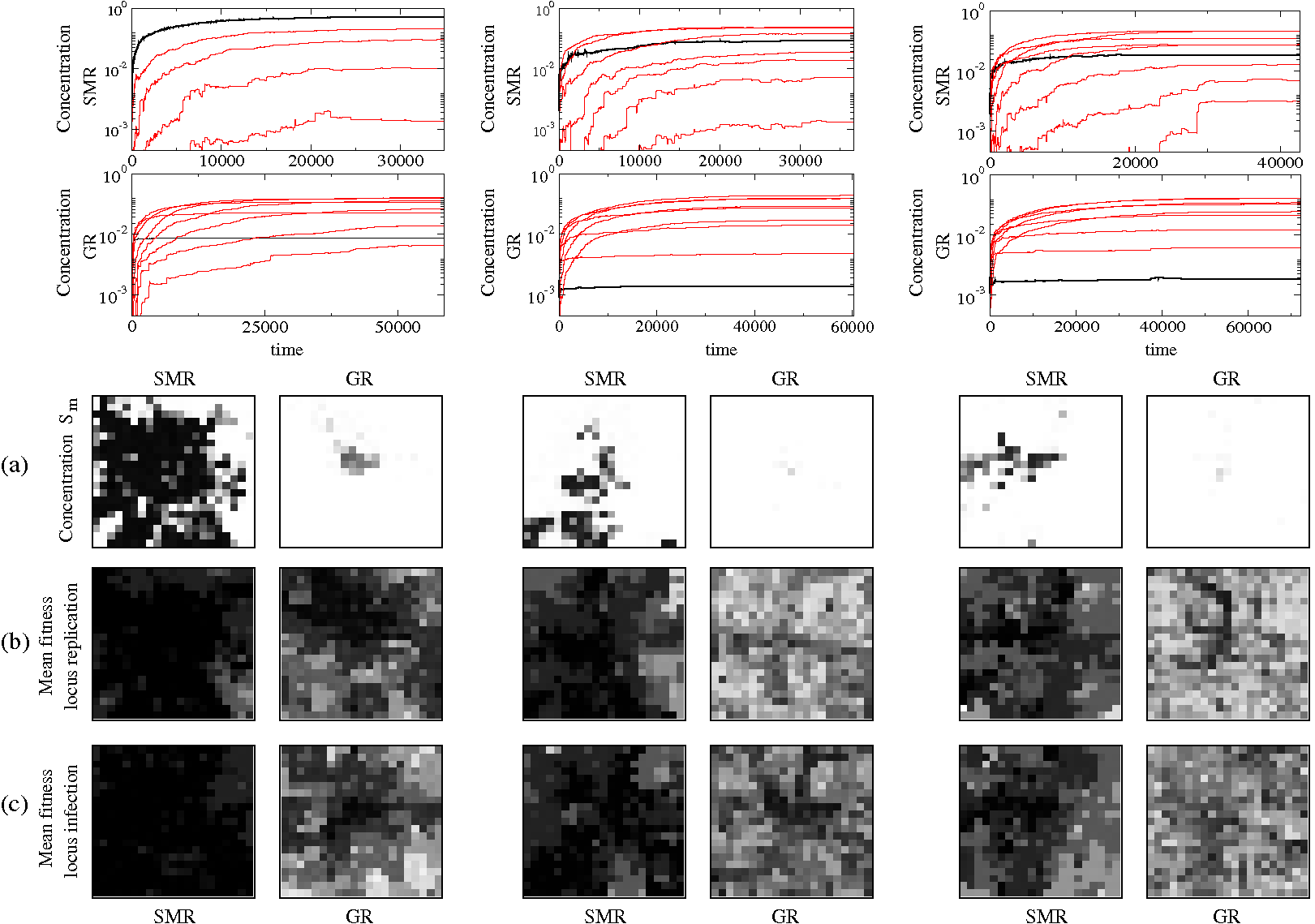

Supplement: Figure S3 — Same as in the previous figure also for the synergistic fitness landscape but now considering FS. (TIFF) [file pone.0024884.s003.tif]

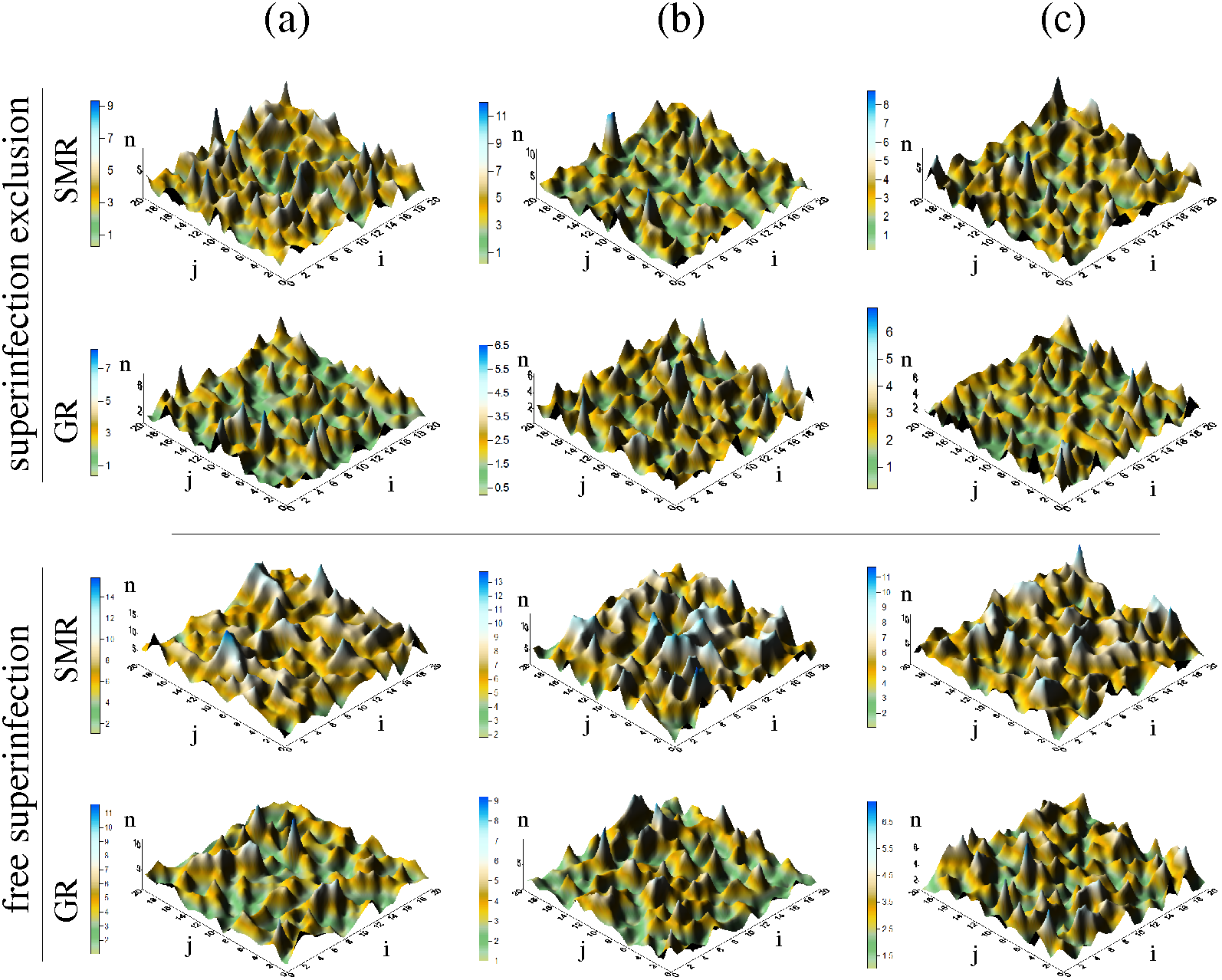

Supplement: Figure S4 — Spatial distribution of the number of infections, (z-axis), in the lattice for a single run under the antagonistic fitness landscape, using (from left to right): (a) , (b) and (c) . We show the spatial pattern for SMR and GR. In the upper and in the lower two rows, we show the spatial patterns considering SE and FS, respectively. Note that these analyses show how does the multiplicity of infection (MOI) dependes on the mode of replication mode and on the fitness landscape, as well as how it distributes in the space. (TIFF) [file pone.0024884.s004.tiff]

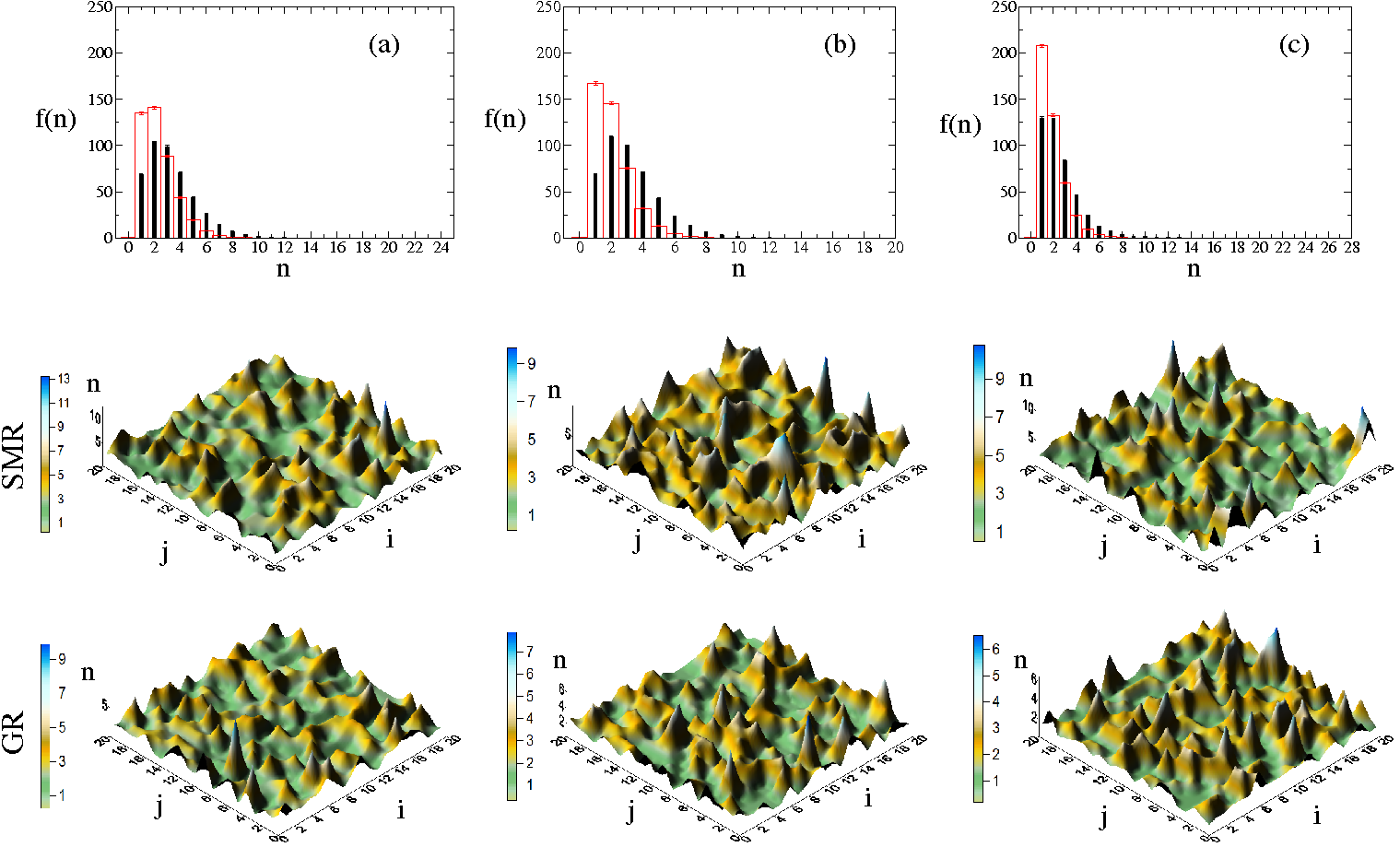

Supplement: Figure S5 — (Upper first row) Absolut frequency distribution, , of the number of cells with infections for the SMR (black histograms) and GR (red histograms) for the synergistic fintess landscape ( ) with SE. Here (a) , (b) and (c) . The histograms correspond to the average ( SEM) number of cells with entering strings computed over independent runs. (Lower two rows) Spatial distribution of the number of infections, (z-axis), in the lattice for a single run for each mutation rate used in (a). We show the results for SMR (upper spaces) and GR (lower spaces). (TIFF) [file pone.0024884.s005.tiff]

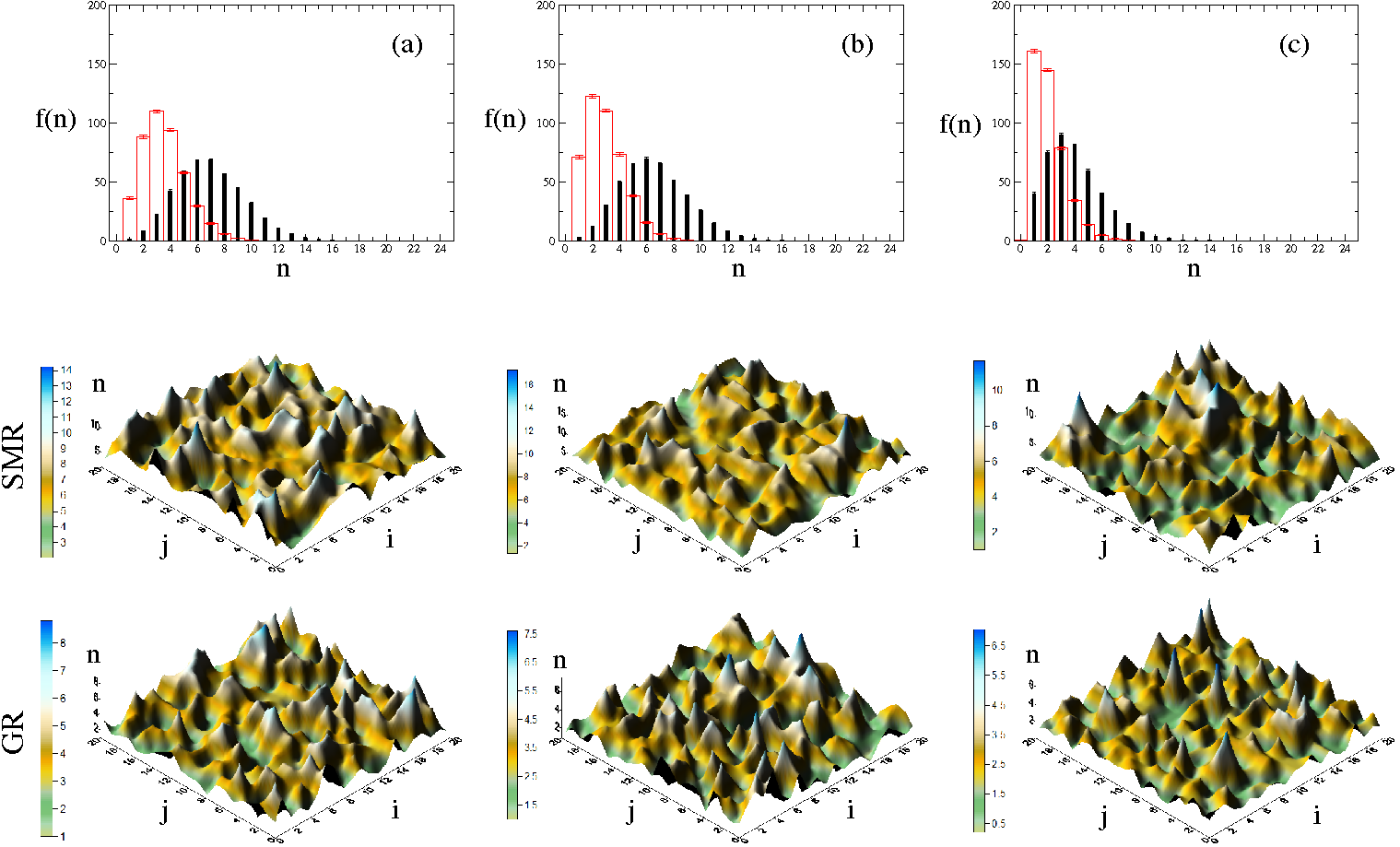

Supplement: Figure S6 — Same as in the previous figure for the synergistic landscape with and FS. (TIFF) [file pone.0024884.s006.tiff]
